# Does tumor size improve the accuracy of prognostic prediction in patients with esophageal squamous cell carcinoma after surgical resection?

**DOI:** 10.18632/oncotarget.11286

**Published:** 2016-08-13

**Authors:** Hongdian Zhang, Peng Tang, Xiaohui Miao, Yongyin Gao, Xiaobin Shang, Lei Gong, Zhao Ma, Mingjian Yang, Hongjing Jiang, Zhongli Zhan, Bin Meng, Zhentao Yu

**Affiliations:** ^1^ Department of Esophageal Cancer, Tianjin Medical University Cancer Institute and Hospital, Key Laboratory of Cancer Prevention and Therapy of Tianjin City, Tianjin 300060, China; ^2^ Department of Thoracic Surgery, Tianjin Haihe Hospital, Tianjin 300350, China; ^3^ Department of Cardiopulmonary Function, Tianjin Medical University Cancer Institute and Hospital, Key Laboratory of Cancer Prevention and Therapy of Tianjin City, Tianjin 300060, China; ^4^ Department of Pathology, Tianjin Medical University Cancer Institute and Hospital, Key Laboratory of Cancer Prevention and Therapy of Tianjin City, Tianjin 300060, China

**Keywords:** esophageal squamous cell carcinoma, tumor size, TNM staging, predictive accuracy, prognosis

## Abstract

This study aimed to investigate whether the inclusion of tumor size could improve the prognostic accuracy in patients with esophageal squamous cell cancer (ESCC). A total of 387 patients with ESCC who underwent curative resection were enrolled in this analysis. The patients were categorized into small-sized tumors (SSTs) and large-sized tumors (LSTs) using an appropriate cut-off point for tumor size. Kaplan–Meier survival curve and log–rank test were used to evaluate the prognostic value of tumor size. A Cox regression model was adopted for multivariate analysis. Their accuracy was compared based on the presence or absence of tumor size. Using 3.5 cm as the optimal cut-off point, 228 and 159 patients presented with LSTs (≥ 3.5 cm) and SSTs (< 3.5 cm), respectively. The patients with LSTs had significantly worse prognoses than patients with SSTs (23.9% vs. 43.2%, *P* < 0.001). Multivariate analysis revealed that tumor size, histological type, invasion depth, and lymph node metastasis were independent predictors of overall survival. The addition of tumor size to the AJCC TNM staging improved the predictive accuracy of the 5-year survival rate by 3.9%. Further study showed that tumor size and T stage were independent predictors of the prognosis of node-negative patients, and the combination of tumor size and T stage improved the predictive accuracy by 3.7%. In conclusion, tumor size is indeed a simple and practical prognostic factor in patients with ESCC. It can be used to improve the prognostic accuracy of the current TNM staging, especially for patients with node-negative disease.

## INTRODUCTION

Esophageal cancer (EC) is a highly common gastrointestinal malignancy with a high incidence worldwide [1]. In China, it is the fifth most frequent cancer and the fourth leading cause of cancer-related deaths [2]. Surgical resection with lymphadenectomy remains the mainstay of potentially curative treatments. However, regardless of the improvements in surgical management and multimodality therapy, the prognosis of EC remains poor. The identification of prognostic factors for EC is extremely important in predicting prognosis and guiding treatment. Factors associated with patient prognosis include invasion depth, lymph node metastasis, histological grading, and tumor location, all of which are included in the newly published American Joint Committee on Cancer (AJCC) TNM staging system [3, 4]. However, these prognostic factors are not available during surgery, which must be confirmed postoperatively.

Tumor size is another important variable referred to as the maximum diameter of primary tumor that can be easily measured before or during surgery without the need of any special technique. It is used to determine a safe surgical margin and the extension of lymphadenectomy in curative esophageal resection [5, 6]. It has been included in the staging systems of many solid tumors, such as lung, breast, and liver cancers [7–9]. However, to date, the variable has not been integrated in the staging of EC, and its clinical value for EC remains elusive. Although tumor size was recently suggested to affect patient survival, no study has assessed its effect on the TNM staging system [10–14].

In this present study, we analyzed the data to elucidate the correlation between tumor size and the prognosis of EC patients after curative resection. We also determined whether the addition of tumor size could improve the prognostic accuracy of the current AJCC TNM staging system.

## RESULTS

### Clinicopathologic features of patients

Among the 387 patients identified, there were 320 (82.7%) men and 67 (17.3%) women. The median age at surgery was 68 (range, 39–99) years. Of these patients, 271 (70.0%) presented with a smoking history, 258 (66.7%) presented with an alcohol consumption history. The most common location of the tumor was in the middle thoracic esophagus (*n* = 314 [81.1%]), followed by the lower thoracic (*n* = 52 [13.4%]) and upper thoracic esophagus (*n* = 21[5.4%]). There were 33 (8.5%) patients well differentiated, 294 (76.0%) cases moderately differentiated, and 60 (15.5%) cases poorly differentiated/undifferentiated. Based on the criteria of the 7th edition AJCC TNM staging system, 42 (10.9%), 36 (9.3%), 222 (57.4%) and 87 (22.5%) cases had pT1, pT2, pT3 and pT4 disease, respectively. Postoperative histological examinations confirmed that lymph node metastasis was present in 164 (42.4%) cases. In addition, 45 cases were classified as stage I tumors, 158 cases were classified as stage II tumors, and 184 cases were classified as stage III tumors by TNM staging.

### Cut-off value of tumor size

The mean ± standard deviation (SD) of tumor size was 4.2 ± 1.9 cm (median: 3.8 cm, range, 0.5–12.0 cm). Regarding the optimal cut-off point for tumor size, the most significant difference in survival was observed at a cut-off point of 3.5 cm, which yielded the largest chi-square value of 22.052 and a hazard ratio (HR) of 1.859 in the Cox proportional hazards model (*P* < 0.001, Supplementary Table S1).

The Youden index (Youden index = sensitivity + specificity − 1) could be defined as a function of sensitivity and specificity, and ranged between 0 and 1. In this measure, values close to 1 indicated relatively large effectiveness. Receiver operating characteristic (ROC) analysis also indicated that a cut-off point of 3.5 cm achieved the maximum Youden index in predicting 5-year survival after surgical resection (Youden's index = 0.373 with a sensitivity of 73.8% and specificity of 63.5%, area under the curve [AUC] = 0.711, and 95% confidence interval (CI) = 0.651–0.772, *P*<0.0001) (Supplementary Figure S1). Given this optimal cut-off value, all patients were divided into two subgroups as follows: 159 (41.1%) patients with small-sized tumors (SSTs, tumor size ≤ 3.5 cm) and 228 (58.9%) patients with large-sized tumors (LSTs, tumor size > 3.5 cm). The clinicopathologic features and prognostic differences between the patients with SSTs and LSTs were reviewed.

### Correlation analysis of tumor size with clinicopathologic variables

Table 1 shows the correlations between tumor size and other clinicopathologic variables. Tumor size was significantly related to invasion depth (*x*^2^ = 14.307, *P* = 0.003), lymph node metastasis (*x*^2^ = 9.478, *P* = 0.024), and advanced TNM staging (*x*^2^ = 7.943, *P* = 0.019). The mean number of metastatic lymph nodes was greater in patients with LSTs than in patients with SSTs (*t* = −2.663, *P* = 0.008). However, gender, age, smoking history, alcohol consumption history, tumor location, and histological type were not statistically associated with tumor size.

**Table 1:** Correlation between tumor size and clinicopathologic features in the patients who underwent curative resection for esophageal cancer (*n* = 387)

Clinicopathologic features	Cases	Tumor size		x^2^ value	*P* value
		SST	LST		
	387	159 (41.1%)	228 (58.9%)		
Gender				1.492	0.222
Male	320	127 (39.7%)	193 (60.3%)		
Female	67	32 (47.8%)	35 (52.2%)		
Age (years)				3.440	0.064
≤ 65	180	65 (36.1%)	115 (63.9%)		
> 65	207	94 (45.4%)	113 (54.6%)		
Smoking history				2.045	0.153
None	116	54 (46.6%)	62 (53.4%)		
Yes	271	105 (38.7%)	166 (61.3%)		
Alcohol consumption history				1.201	0.273
None	129	58 (45.0%)	71 (55.0%)		
Yes	258	101 (39.1%)	157 (60.9%)		
Tumor location				3.639	0.162
Upper	21	5 (23.8%)	16 (76.2%)		
Middle	314	129 (41.1%)	185 (58.9%)		
Lower	52	25 (48.1%)	27 (51.9%)		
Histological type				1.206	0.547
G1	33	13 (39.4%)	20 (60.6%)		
G2	294	125 (42.5%)	169 (57.5%)		
G3	60	21 (35.0%)	39 (65.0%)		
Invasion depth				14.307	0.003
pT1	42	17 (40.5%)	25 (59.5%)		
pT2	36	22 (61.1%)	14 (38.9%)		
pT3	222	97 (43.7%)	125 (56.3%)		
pT4	87	23 (26.4%)	64 (73.6%)		
Lymph node metastasis				9.478	0.024
pN0	223	101 (45.3%)	122 (54.7%)		
pN1	99	42 (42.4%)	57 (57.6%)		
pN2	35	10 (28.6%)	25 (71.4%)		
pN3	30	6 (20.0%)	24 (80.0%)		
TNM staging				7.943	0.019
I	45	22 (48.9%)	23 (51.1%)		
II	158	75 (47.5%)	83 (52.5%)		
III	184	62 (33.7%)	122 (66.3%)		

Abbreviation: SST, small sized tumor; LST, large sized tumor; TNM, Tumor-nodes metastasis;

G1, well differentiated; G2, moderately differentiated; G3, poorly differentiated/undifferentiated.

### Univariate and multivariate survival analyses for all patients

The median follow-up period for the entire cohort was 30 months (range, 3 −108 months). The cumulative 1-, 3-, and 5-year survival rates for all the patients were 78.6%, 42.4%, and 31.2%, respectively, with the median survival time (MST) was 29.5 months.

**Figure 1:**
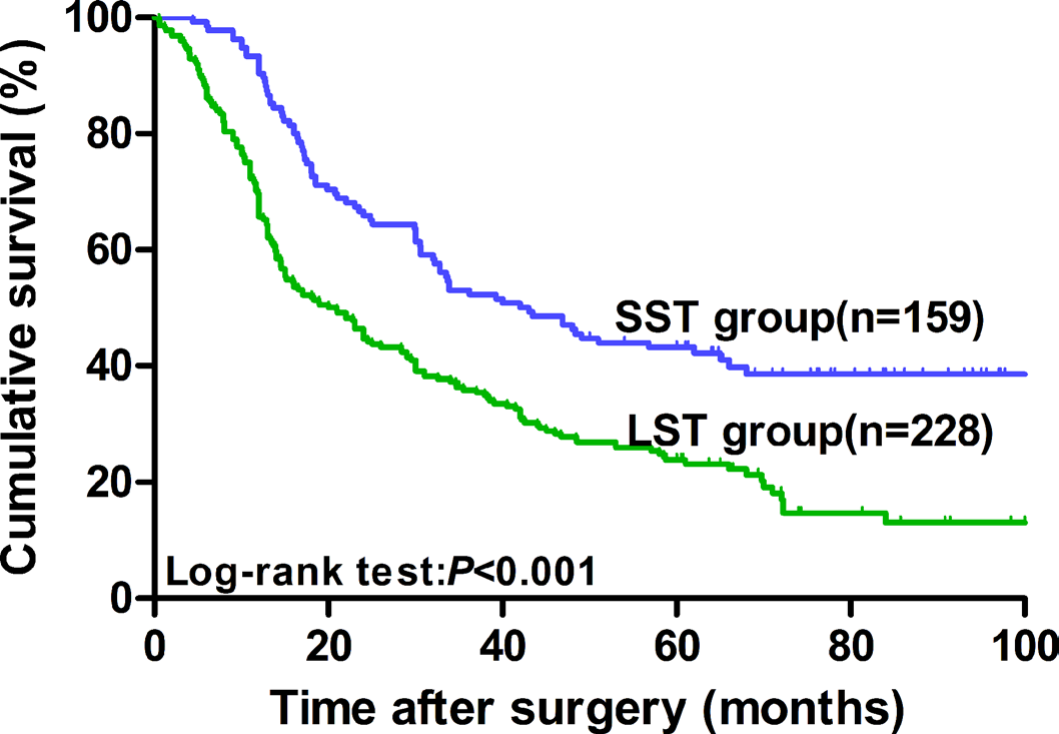
Kaplan–Meier survival curves in ESCC patients who underwent curative esophagectomy according to tumor size (*n* = 387) The prognosis of LST patients was significantly worse than that of SST patients (23.9% vs. 43.2%, *P* < 0.001).

As shown in Figure 1, the 5-year survival rate for SST patients was 43.2% (MST was 43.0 months), whereas that for LST patients was 23.9% (MST was 20.0 months). Thus, a statistically significant difference was observed (*x^2^* = 24.204, *P* < 0.001).

To determine whether tumor size was an independent factor associated with overall survival (OS) in esophageal squamous cell cancer (ESCC) patients, univariate Kaplan–Meier analysis was performed to assess the predictive capability of each variable. As shown in Table 2, patient age (*P* = 0.038), smoking history (*P* = 0.026), tumor size (*P* < 0.001), histological type (*P* = 0.036), invasion depth (*P* < 0.001), and lymph node metastasis (*P* < 0.001) were significant factors associated with OS in the entire patient population. By contrast, no significant difference in gender (*P* = 0.558), alcohol consumption history (*P* = 0.073), and tumor location (*P* = 0.171) was noted.

**Table 2:** Univariate analysis of various clinicopathologic features for overall survival by Kaplan–Meier method (log-rank test)

Clinicopathologic features	Cases	5-YSR (%)	Median survival time (months)	x^2^ value	*P* value
Gender				0.343	0.558
Male	320	31.5	29.5		
Female	67	29.4	29.0		
Age (years)				4.290	0.038
≤ 65	180	34.2	33.5		
> 65	207	28.6	23.0		
Smoking history				4.967	0.026
None	116	39.9	42.0		
Yes	271	27.5	24.0		
Alcohol consumption history				3.212	0.073
None	129	37.2	33.9		
Yes	258	28.2	24.8		
Tumor location				3.536	0.171
Upper	21	23.8	20.0		
Middle	314	30.1	25.0		
Lower	52	40.8	44.0		
Tumor size				24.204	0.000
SST	159	43.2	43.0		
LST	228	23.9	20.0		
Histological type				6.625	0.036
G1	33	49.2	57.0		
G2	294	30.8	29.5		
G3	60	22.5	17.0		
Invasion depth				28.118	0.000
pT1	42	57.9	NA		
pT2	36	36.2	38.3		
pT3	222	29.4	30.0		
pT4	87	21.3	13.5		
Lymph node metastasis				68.800	0.000
pN0	223	42.5	43.0		
pN1	99	21.9	23.0		
pN2	35	7.1	15.0		
pN3	30	3.4	10.0		

Abbreviation: 5-YSR, 5-year survival rate; SST, small sized tumor; LST, large sized tumor; NA, not available.

The six variables for OS with prognostic potential were subsequently subjected to multivariate analysis using the Cox proportional hazards model. Tumor size (*P* < 0.001, HR = 1.703), histological type (*P* = 0.032, HR = 1.321), invasion depth (*P* < 0.001, HR = 1.359), and lymph node metastases (*P* < 0.001, HR = 1.513) independently predicted poor prognosis for the entire population. However, age and smoking history, which were significant prognostic factors in univariate analysis, did not independently influence patient survival in multivariate analysis. Compared with the SST patients, the LST patients held a 1.703 increased risk of death (95%CI = 1.296–2.239, *P* < 0.001) (Table 3).

**Table 3:** Multivariate survival analysis of prognostic features by Cox regression model

Clinicopathologic features	B	Wald	*P* value	HR	95%CI
Age	0.230	3.065	0.080	1.258	0.973 − 1.627
Smoking history	0.262	3.279	0.070	1.299	0.979 − 1.725
Tumor size	0.533	14.586	**0.000**	1.703	1.296 − 2.239
Histological type	0.278	4.595	**0.032**	1.321	1.024 − 1.703
Invasion depth	0.307	13.894	**0.000**	1.359	1.157 − 1.597
Lymph node metastasis	0.414	41.422	**0.000**	1.513	1.334 − 1.717

Abbreviation: HR, hazard ratio; CI, confidence interval.

### Impact of tumor size on 5-year postoperative survival rate with the TNM staging system

Calculations using the 7th AJCC TNM staging system revealed that, the accuracies of invasion depth and lymph node metastasis in predicting 5-year survival rates were 69.1% and 71.3%, respectively. Meanwhile, the accuracy of the 7th AJCC TNM staging system in predicting 5-year survival rate was 72.3%, whereas the 5-year survival rate predicted by a combination of tumor size and the 7th AJCC TNM staging system was 76.2%, resulting in a median increase of 3.9% (*P* < 0.05, Table 4).

**Table 4:** Univariate and multivariate Cox regression models predicting invasion depth, lymph node metastasis, tumor size and 5-year survival, according to 7th AJCC TNM staging in ESCC patients

Variables	5-year survival				
	Univariate		Multivariate (T+N+M0)		Multivariate (T+N+M0+S)
	HR *P* value (95% CI)	Predictive accuracy of univariate	HR *P* value (95% CI)		HR *P* value (95% CI)
T stage (T)	–	0.691	–		−
T2 vs. T1	0.277		0.288		0.299
	0.000		0.000		0.000
	0.16 – 0.479		0.166 – 0.501		0.171 − 0.520
T3 vs. T1	0.477		0.547		0.623
	0.003		0.016		0.032
	0.295 – 0.771		0.335 – 0.892		0.379 − 0.724
T4 vs. T1	0.634		0.622		0.649
	0.002		0.001		0.004
	0.476 – 0.844		0.464 – 0.833		0.482 − 0.873
N stage (N)	–	0.713	−		−
N1 vs. N0	0.228		0.224		0.248
	0.000		0.000		0.000
	0.150 – 0.346		0.147 – 0.343		0.162 − 0.380
N2 vs. N0	0.400		0.405		0.437
	0.000		0.000		0.000
	0.257 – 0.622		0.259 − 0.633		0.279 − 0.684
N3 vs. N0	0.607		0.492		0.504
	0.000		0.008		0.011
	0.362 – 0.817		0.291 − 0.833		0.297 − 0.855
Tumor size (S)	0.521	0.707	−		0.588
	0.000				0.000
	0.400 – 0.680				0.449 − 0.771
Predictive accuracy of the model			0.723	0.762	(+3.9%)

Abbreviation: S, tumor size; HR hazard ratio; CI, confidence interval.

### Stage-stratified analysis of patient survival

**Figure 2:**
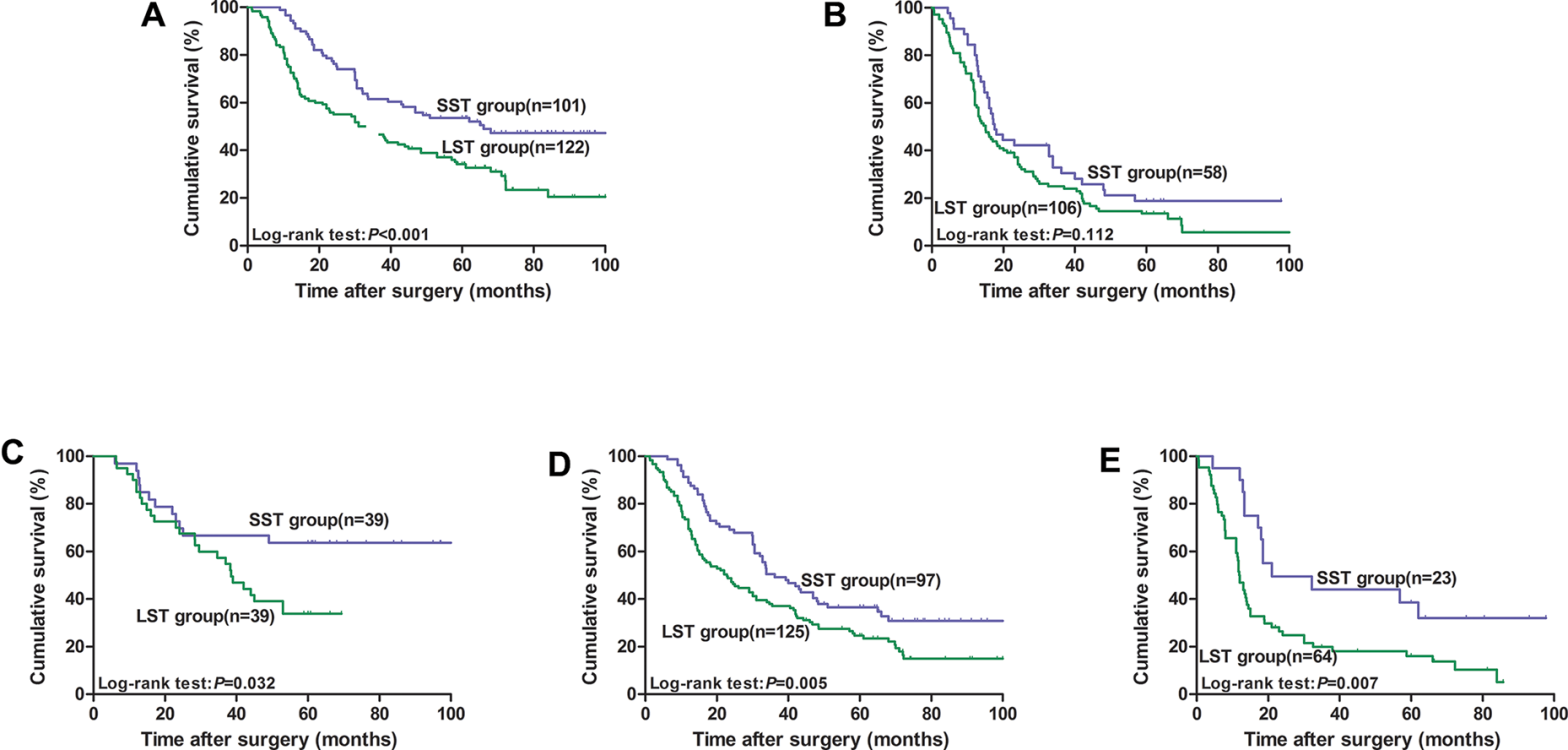
Kaplan–Meier survival curves for ESCC patients stratified by lymph node status and invasion depth after curative esophagectomy according to tumor size (**A**) For node-negative patients; (**B**) For node-positive patients; (**C**) For T1–2 patients; (**D**) For T3 patients; (**E**) For T4 patients.

To eliminate the confounding effect of tumor size on lymph node status (node negative or positive) and invasion depth (T1–2, T3, and T4), we further performed a stage-stratified analysis of all patients in accordance with the current TNM staging system. As shown in Figure 2, for the patients without lymph node metastasis (*n* = 223), the survival of LST patients was worse than that of SST patients (5-YSR: 53.5% vs. 34.2%, *P* < 0.001, Figure 2A). However, the 5-year OS rate of patients with SSTs were similar to those of LST patients (5-YSR: 18.8% vs. 13.5%, *P* = 0.112, Figure 2B) when lymph nodes were involved (*n* = 164). For the patients at stage T1–2 (*n* = 78), the 5-year OS rate of patients with SSTs was significantly higher than that of patients with LSTs (5-YSR: 63.6% vs. 33.9%, *P* = 0.032, Figure 2C). For the patients at stage T3 (*n* = 222) and T4 (*n* = 87), the 5-year OS rates of patients with SSTs were also significantly higher than that of patients with LSTs (For T3 cases, 5-YSR: 36.5% vs. 24.7%, *P* = 0.005, Figure 2D; For T4 cases, 5-YSR:38.5% vs. 16.0%, *P* = 0.007, Figure 2E).

### Predictive accuracy of the combination of T stage and tumor size for patients without lymph node metastasis

To obtain a more comprehensive understanding of the clinical significance of tumor size for node-negative patients, univariate and multivariate survival analyses were taken to determine potential prognostic factors that affect the outcome. As shown in Table 5, statistical significance was obtained in age, tumor size, histological type, and invasion depth according to univariate analysis on prognosis. Meanwhile, multivariate analysis using the Cox proportional hazards model confirmed tumor size (*P* = 0.001, HR = 1.878) and invasion depth (*P* = 0.018, HR = 1.295) as independent prognostic factors.

**Table 5:** Univariate and multivariate survival analyses of clinicopathologic variables for patients without lymph node metastasis (*n* = 164)

Variable	Univariate analysis			Multivariate analysis			
	HR	95% CI	*P* value	Wald	HR	95% CI	*P* value
**Gender** Male/Femal	1.089	0.688 − 1.724	0.716				
**Age (years)** ≤ 65 / > 65	1.566	1.050 − 2.336	**0.028**	0.852	1.188	0.824 − 1.713	0.356
**Smoking history** None/Yes	1.245	0.881 − 1.761	0.214				
**Alcohol consumption history** None/Yes	1.477	0.991 − 2.203	0.056				
**Tumor location** Upper/Middle/Lower	0.769	0.511 − 1.156	0.207				
**Tumor size** SST/LST	1.908	1.328 − 2.742	**0.000**	11.231	1.878	1.299 − 2.715	**0.001**
**Histological type** G1/G2/G3	1.512	1.082 − 2.112	**0.015**	3.683	1.381	0.993 − 1.920	0.055
**Invasion depth** pT1/pT2/pT3/pT4	1.369	1.107 − 1.693	**0.004**	5.571	1.295	1.045 − 1.604	**0.018**

Abbreviation: SST, small sized tumor; LST, large sized tumor; HR, hazard ratio; CI, confidence interval.

Furthermore, the accuracy of invasion depth alone in predicting the 5-year survival rate was 68.1%, whereas the addition of tumor size to invasion depth significantly improved the accuracy of prediction to 71.8%, with an increase of 3.7% in accuracy (*P* < 0.05, Table 6).

**Table 6:** Univariate and multivariate Cox regression models predicting invasion depth, tumor size and 5-year survival in ESCC patients without lymph node metastasis

Variables	5-year survival		
	Univariate		Multivariate (T+S)
	HR *P* value (95% CI)	Predictive accuracy of univariate	HR *P* value (95% CI)
T stage (T)		0.681	
T2 vs. T1	0.373		0.387
	0.007		0.010
	0.181 − 0.566		0.188 − 0.795
T3 vs. T1	0.582		0.698
	0.036		0.028
	0.302 − 0.774		0.360–0.855
T4 vs. T1	0.769		0.840
	0.028		0.047
	0.502 − 0.879		0.547−0.791
Tumor size (S)	0.524	0.714	0.529
	0.000		0.001
	0.365 − 0.753		0.367 − 0.764
Predictive accuracy of the model			0.718 (+3.7%)

Abbreviation: S, tumor size; HR, hazard ratio; CI, confidence interval.

### ROC analyses

**Figure 3:**
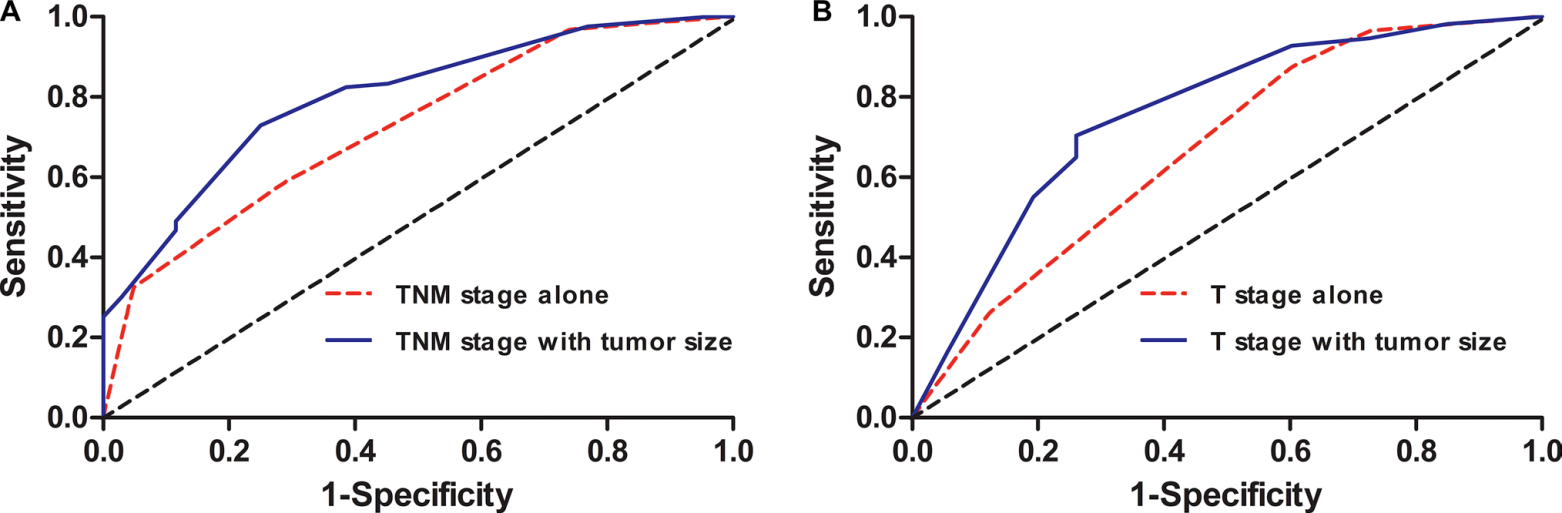
ROC curves used to evaluate the predictive accuracy of 5-year survival rates (**A**) TNM stage alone and combined with tumor size for all patients; (**B**) T stage alone and combined with tumor size for node-negative patients.

Finally, the AUC based on ROC curves for 5-year OS were measured and compared using the method established by DeLong et al. The prediction ability of the conventional AJCC TNM staging system was 0.728 (95% CI = 0.665–0.781), and the combination of TNM staging system with tumor size improved the prognostic ability from 0.728 to 0.796 (95% CI = 0.749–0.849) for all patients (*P* < 0.05, Figure 3A).

Similarly, the use of invasion depth combined with tumor size (AUC = 0.745, 95% CI = 0.684–0.830) was significantly superior to the use of invasion depth alone (AUC = 0.679, 95% CI = 0.589–0.752) in predicting 5-year survival for node-negative patients (*P* < 0.05, Figure 3B).

## DISCUSSION

EC is an extremely aggressive malignancy with increasing incidence in recent years. A uniform and accurate disease staging system of patients is essential to assess appropriate treatment modalities and predict prognosis [15].

Tumor size is an easily measured variable before or during surgery, which has been used as the T stage for many solid tumors, such as lung, breast, thyroid, uterine, and liver cancers [7–9]. Before 1987, esophageal tumor length ≤ 5 cm was categorized as T1 status and > 5 cm as T2 status by the 1983 version TNM staging system. However, tumor length was omitted and replaced by depth of the esophageal wall invasion in the 1987 version of the tumor staging system, and this modification has continued to the present [4, 16, 17].

Recently, the prognostic value of tumor size in EC has received increased attention again and constantly pointed out by several previous studies as a non-negligible prognostic indicator that determines the survival of EC [10–14]. However, no consensus has been reached about the role of tumor size in the disease because of the limitations in sample size, evaluation criteria, and variety of cut-off values.

Although several authors have indicated the superiority of tumor size in EC, the definition of significant prognostic cut-off point varies among studies. Eloubeidi et al. [18] analyzed the outcome of 10,441 EC patients from the database of the National Cancer Institute Surveillance, Epidemiology and End Results data to identify the prognostic factors of tumor size in patients with localized disease. They demonstrated that tumor size greater than 3 cm was associated with decreased OS when compared with shorter tumors. They also suggested that the T status could be suffixed with either an “a” (≤ 3 cm) or “b” (> 3 cm) to modify the current TNM staging. Meanwhile, Griffiths et al. [10] and Yendamuri et al. [19] retrospectively evaluated 309 and 209 EC patients after esophagectomy, and concluded that tumor size was an independent predictor of long-term survival for adenocarcinomas, except for ESCC when classified at a cut-off of 3.5 cm. However, these two studies had difficulty in reaching a consistent conclusion based on such small numbers of patients with ESCC. Zeybek et al. [5] categorized the maximum diameter of tumor into three subgroups (≤ 3 cm, 3–6 cm, and ≥ 6 cm), and they found that both the OS and disease-free survival rates decreased with the increase in tumor size, especially in ESCC. In another study of patients with early EC, researchers found that esophageal tumor size (> 3 cm) in combination with submucosal involvement may help identify the high-risk group of patients for adjuvant therapy and more extensive lymphadenectomy [20].

In the present study, two methods were adopted to determine the optimal cut-off value for tumor size. In the first model, 0.5 cm was set as the standard interval, and 19 cut-off points were checked one by one. After evaluating the two groups by log-rank test, we identified two subgroups of patients with remarkably different survival rates as follows: ≤ 3.5 and > 3.5 cm. In the second method, as shown by the ROC curve, the cut-off point of 3.5 cm for tumor size could provide the best compromise between specificity and sensitivity. Therefore, we adopted a 3.5 cm cut-off value for tumor size in the succeeding study.

Subsequent analysis found that larger tumors were usually closely associated with a greater degree of malignancy and worse biological behavior. Furthermore, the patients with LSTs presented with worse prognosis than the patients with SSTs, which may be attributed to the former's aggressive features. In particular, tumor size was further demonstrated by multivariate Cox regression analysis as an independent prognostic predictor along with invasion depth, lymph node metastasis, and histological type. This research suggested that tumor size could provide important information on malignant potential of tumors and patient outcomes. Therefore, it should be included in the current TNM staging system to enhance the prediction of patient prognosis.

As we all known, the AJCC TNM staging system is important for assessing the prognosis of EC patients. Although tumor size was not included in the new staging system, our study verified that combination of tumor size and the TNM staging could enhance the accuracy of the AJCC TNM staging system alone in predicting the 5-year survival rate among ESCC patients who underwent curative surgery. As a result, tumor size may become another important indicator in the future AJCC TNM staging system.

Some possible mechanisms could explain the relationship between the tumor size and prognosis in ESCC patients. Firstly, the maximum diameter of tumor could provide important information useful for evaluating the potential effect of tumor doubling time on screening programs in terms of the degree of prognostic improvement. Secondly, the great association of a larger tumor size with a more intensive invasion depth, higher incidence of lymph node metastasis, and advanced TNM staging may reflect the value of tumor size as an indicator of aggressive biological behavior of ESCC. Moreover, a large diameter of primary tumor was frequently characterized by histologically poorly differentiated type, which may account for its independent clinical value.

Notably, we found that invasion depth and lymph node involvement were powerful prognostic indicators. To eliminate any confounding effects on OS, we performed a stage-stratified analysis of all patients according to lymph node status and invasion depth. Our results showed that the SST groups presented an overwhelming survival advantage over the LST groups for the lymph node-negative rather than the lymph node-positive patients, which is consistent with previous studies [19, 21]. Moreover, the predictive value was significant for the T1–2, T3, and T4 tumors. Furthermore, tumor size was further demonstrated as an independent prognostic factor, and the inclusion of tumor size could improve predictive accuracy in patients assorted by T stage for node-negative ESCC patients.

However, our study had several potential limitations. It was a retrospective study in nature with a relatively small sample population, leaving some groups small in the statistical analyses. In addition, our data originated from a single institution but with different surgeons and pathologists. Furthermore, a standardized guideline and regimen for postoperative therapy was lacking during the period of this study. Because of this deficiency, we did not evaluate the potential survival benefit possibly related to adjuvant therapy. Further investigations should be performed involving a larger sample size, randomized prospective cohorts, and practical methods from multicenter institutions to determine the optimal cut-off point and confirm these preliminary results in the future.

In conclusion, we showed that the histologically determined 3.5 cm may be the optimal cut-off point for tumor size. It provided important information on tumor aggressiveness and was an independent risk factor correlated with long-term survival in patients with ESCC, especially in node-negative cases. The measurement of tumor size may be a non-negligible criterion for improving the accuracy of prognostic prediction and identifying patients at high risk who would be candidates for additional preoperative or postoperative therapy.

## MATERIALS AND METHODS

### Patients

A consecutive series of 387 patients with histopathologically confirmed ESCC who underwent curative esophagectomy with standard lymphadenectomy at Tianjin Medical University Cancer Institute and Hospital from January 2005 to December 2009 were retrospectively analyzed. Curative resection was defined as complete tumor removal with no macroscopic residual tumor, no invasion of carcinoma cell at any margin, and no evidence of distant metastasis. The inclusion criteria were as follows: (1) tumors located in the thoracic esophagus, (2) tumors confirmed as squamous cell carcinoma, (3) with no history of neoadjuvant radiotherapy or chemotherapy, (4) underwent radical esophagectomy and lymph node dissection, (5) with negative surgical margin, and (6) staging of EC as pT1–4aN0–3M0 based on the 7th edition AJCC TNM staging system.

All patients were evaluated by esophagogastroscopy, barium meal, computed tomography (CT) of the chest and abdomen, and ultrasound of the neck and retroperitoneal lymph nodes for preoperative staging. None of these patients presented with distant metastasis. Cardiac and pulmonary function examinations and other blood tests were also performed to assess surgical tolerance.

This retrospective study protocol was approved by the Research Ethics Committee of Tianjin Medical University Cancer Institute and Hospital, China, and written informed consent was obtained from all patients.

### Surgical approaches and pathological examination

An open Ivor–Lewis transthoracic esophagectomy with two-field lymph node dissection was performed, and a gastric conduit was used as reconstruction substitute in all patients. The thoracic operation consisted of a radical mediastinal dissection, which included resection of bilateral mediastinal pleurae, aortic adventitia, and mediastinal lymphadenectomy. A segment of the pericardium was not routinely resected. In the abdominal phase, dissection of paracardial nodes and enlarged celiac axis nodes (including celiac nodes, left gastric nodes, common hepatic nodes, and splenic nodes) were performed.

Then, all the removed tumor specimens and retrieved lymph nodes were sent fresh for pathology examination by two pathologists, at least one being a specialist upper gastrointestinal pathologist. We took photographs and drew the appearance of all the resected specimens, and made detailed records of them, including tumor size and the cut margins. Tumor size was measured in accordance with the following procedure. First, the resected esophagus was cut open along the longitudinal axis to observe the entire mucosa. The specimen was then placed on a flat table without any stretching assistance. The longitudinal diameter of each specimen was examined with a hand-held ruler and recorded in the pathologic report. The distances between the tumor border and both the proximal and distal cut ends were also recorded. In our study, all the surgical margins were confirmed negative by pathological examination.

Histological type was defined as well differentiated, moderately differentiated, or poorly differentiated/undifferentiated, according to the World Health Organization classification of esophageal tumors [22]. Pathologic staging of the tumor was performed based on the 7th edition of the AJCC TNM staging system for EC [4].

### Classification of tumor size

To facilitate the use of tumor size in clinical practice, the tumor size must be transformed from a continuous variable to a categorical variable by an appropriate cut-off point. In the present study, two methods were used to determine the optimal cut-off point for tumor size in the prediction of the 5-year survival. Initially, the Cox proportional hazards model was used to select the appropriate cut-off value by comparing survival rates between different size groups (at each 0.5 cm interval). The threshold value for tumor size with the largest chi-square value was considered the optimal cut-off point [23]. Subsequently, an ROC curve was plotted, from which the AUC was used as an estimation of the predictive accuracy. The Youden index, which was calculated using the formula [1 − (false positive rate + false negative rate)], was applied on the data [24]. This index corresponded to the optimal cut-off, defined as the value with the highest average sensitivity and specificity [25].

### Follow up

After curative surgery, all of the patients were followed up by hospital visit, mail, or telephone call every 3 months for the first year, every 6 months for the second year, and then every year until death or the last follow up. The deadline of the follow-up was set at December 2014, and the follow-up rate was 90.7%. The cases lost to follow up were treated as censored data for the analysis of survival. A total of 258 patients (66.7%) died during the follow-up period. OS time was determined from the date of surgery to the date of death or the last follow up.

### Statistical analysis

Statistical analyses were performed using SPSS 17.0 (SPSS, Inc., Chicago, IL) software and the R (http://www.r-project.org/) software. All patients included in our study were retrospectively evaluated by gender, age, smoking history, alcohol consumption history, tumor location, tumor size, histological type, invasion depth, lymph node metastasis, and TNM staging. Measurement data were presented as the mean ± SD. The correlation between categorical variables was determined using Pearson *x*^2^ or Fisher's exact tests. Survival analysis was performed using the Kaplan–Meier method and valided by the log-rank test. The significant clinicopathologic factors were included into the Cox proportional hazards regression model to determine the independent prognostic factors and compute their HRs and 95% CIs.

Furthermore, Cox regression models addressed the OS, where T stage, N stage and M stage were complemented with tumor size. Each model was then subjected to bootstrap resampling for internal validation and to reduce overfit bias. Predictive accuracy estimates, as defined by Harrell, were then compared between those models based on whether they include tumor size or not [26–28]. Finally, The ROC curves were plotted to illustrate the role of tumor size in improving the stage predictive accuracy. Comparisons between the AUCs were accomplished with the nonparametric approach established by DeLong et al. [29]. A two-tailed *P* value of < 0.05 was considered statistically significant.

## SUPPLEMENTARY MATERIAL FIGURE AND TABLE



## References

[1] Siegel R, Ma J, Zou Z, Jemal A (2014). Cancer statistics, 2014. CA Cancer J Clin.

[2] Chen W, Zheng R, Zhang S, Zhao P, Li G, Wu L, He J (2013). The incidences and mortalities of major cancers in China, 2009. Chin J Cancer.

[3] Wittekind C (2010). 2010 TNM system: on the 7th edition of TNM classification of malignant tumors. Pathologe.

[4] Edge SB, Compton CC (2010). The American Joint Committee on Cancer: the 7th edition of the AJCC cancer staging manual and the future of TNM. Ann Surg Oncol.

[5] Zeybek A, Erdogan A, Gulkesen KH, Ergin M, Sarper A, Dertsiz L, Demircan A (2013). Significance of tumor length as prognostic factor for esophageal cancer. Int Surg.

[6] Chao YK, Tseng CK, Wen YW, Liu YH, Wan YL, Chiu CT, Chang WC, Chang HK (2013). Using pretreatment tumor depth and length to select esophageal squamous cell carcinoma patients for nonoperative treatment after neoadjuvant chemoradiotherapy. Ann Surg Oncol.

[7] Wittekind C (2014). New TNM classification of lung tumors. Pathologe.

[8] Schwartz AM, Henson DE, Chen D, Rajamarthandan S (2014). Histologic grade remains a prognostic factor for breast cancer regardless of the number of positive lymph nodes and tumor size: a study of 161 708 cases of breast cancer from the SEER Program. Arch Pathol Lab Med.

[9] Duseja A (2014). Staging of hepatocellular carcinoma. J Clin Exp Hepatol.

[10] Griffiths EA, Brummell Z, Gorthi G, Pritchard SA, Welch IM (2006). Tumor length as a prognostic factor in esophageal malignancy: univariate and multivariate survival analyses. J Surg Oncol.

[11] Wu N, Pang LW, Chen ZM, Ma QY, Chen G (2012). Tumour length is an independent prognostic factor of esophageal squamous cell carcinomas. Chin Med J (Engl).

[12] Wang BY, Liu CY, Lin CH, Hsu PK, Hsu WH, Wu YC, Cheng CY (2012). Endoscopic tumor length is an independent prognostic factor in esophageal squamous cell carcinoma. Ann Surg Oncol.

[13] Feng JF, Huang Y, Zhao Q (2013). Tumor length in elderly patients with esophageal squamous cell carcinoma: is it a prognostic factor?. Ups J Med Sci.

[14] Bollschweiler E, Baldus SE, Schroder W, Schneider PM, Holscher AH (2006). Staging of esophageal carcinoma: length of tumor and number of involved regional lymph nodes. Are these independent prognostic factors?. J Surg Oncol.

[15] Strong VE, D'Amico TA, Kleinberg L, Ajani J (2013). Impact of the 7th Edition AJCC staging classification on the NCCN clinical practice guidelines in oncology for gastric and esophageal cancers. J Natl Compr Canc Netw.

[16] Iizuka T, Isono K, Kakegawa T, Watanabe H (1989). Parameters linked to ten-year survival in Japan of resected esophageal carcinoma. Japanese Committee for Registration of Esophageal Carcinoma Cases. Chest.

[17] Sobin LH, Hermanek P, Hutter RV (1988). TNM classification of malignant tumors. A comparison between the new (1987) and the old editions. Cancer.

[18] Eloubeidi MA, Desmond R, Arguedas MR, Reed CE, Wilcox CM (2002). Prognostic factors for the survival of patients with esophageal carcinoma in the U.S.: the importance of tumor length and lymph node status. Cancer.

[19] Yendamuri S, Swisher SG, Correa AM, Hofstetter W, Ajani JA, Francis A, Maru D, Mehran RJ, Rice DC, Roth JA, Walsh GL, Vaporciyan AA (2009). Esophageal tumor length is independently associated with long-term survival. Cancer.

[20] Bolton WD, Hofstetter WL, Francis AM, Correa AM, Ajani JA, Bhutani MS, Erasmus J, Komaki R, Maru DM, Mehran RJ, Rice DC, Roth JA, Vaporciyan AA (2009). Impact of tumor length on long-term survival of pT1 esophageal adenocarcinoma. J Thorac Cardiovasc Surg.

[21] Wang BY, Goan YG, Hsu PK, Hsu WH, Wu YC (2011). Tumor length as a prognostic factor in esophageal squamous cell carcinoma. Ann Thorac Surg.

[22] Flejou JF (2011). WHO Classification of digestive tumors: the fourth edition. Ann Pathol.

[23] Guo P, Li Y, Zhu Z, Sun Z, Lu C, Wang Z, Xu H (2013). Prognostic value of tumor size in gastric cancer: an analysis of 2,379 patients. Tumor Biol.

[24] Alemayehu D, Zou KH (2012). Applications of ROC analysis in medical research: recent developments and future directions. Acad Radiol.

[25] Wang Y, Zhao H, Gao X, Wei F, Zhang X, Su Y, Wang C, Li H, Ren X (2016). Identification of a three-mirna signature as a blood-borne diagnostic marker for early diagnosis of lung adenocarcinoma. Oncotarget.

[26] Harrell FJ, Lee KL, Mark DB (1996). Multivariable prognostic models: issues in developing models, evaluating assumptions and adequacy, and measuring and reducing errors. Stat Med.

[27] Karakiewicz PI, Lewinshtein DJ, Chun FK, Briganti A, Guille F, Perrotte P, Lobel B, Ficarra V, Artibani W, Cindolo L, Tostain J, Abbou CC, Chopin D (2006). Tumor size improves the accuracy of TNM predictions in patients with renal cancer. Eur Urol.

[28] Lu J, Huang CM, Zheng CH, Li P, Xie JW, Wang JB, Lin JX (2013). Consideration of tumor size improves the accuracy of TNM predictions in patients with gastric cancer after curative gastrectomy. Surg Oncol.

[29] DeLong ER, DeLong DM, Clarke-Pearson DL (1988). Comparing the areas under two or more correlated receiver operating characteristic curves: a nonparametric approach. Biometrics.

