# Supplementary material for: Does tumor size improve the accuracy of prognostic prediction in patients with esophageal squamous cell carcinoma after surgical resection?
Source: Oncotarget. 2016 Aug 13;7(41):66623–34. doi: 10.18632/oncotarget.11286 (PMC5341825; doi:10.18632/oncotarget.11286)
Supplement: Supplementary file 1 [file oncotarget-07-66623-s001.pdf]

Does tumor size improve the accuracy of prognostic prediction in patients with esophageal squamous cell carcinoma after surgical resection?

Supplementary Materials

Supplementary Table S1: Chi-square values and hazard ratios calculated by the Cox proportional hazards model based on the different cut-off points for tumor size

Cut-off point (cm)	x^2 value	*P* value	HR (95%CI)
1.0	1.976	0.160	4.090 (0.574~29.157)
1.5	3.385	0.066	2.529 (0.941~6.795)
2.0	4.571	0.033	1.596 (1.040~2.451)
2.5	17.206	0.000	2.244 (1.532~3.288)
3.0	17.287	0.000	1.803 (1.365~2.380)
**3.5**	**22.052**	**0.000**	**1.859 (1.428~2.421)**
4.0	9.695	0.002	1.475 (1.155~1.884)
4.5	5.846	0.016	1.356 (1.059~1.737)
5.0	6.211	0.013	1.412 (1.076~1.852)
5.5	6.772	0.009	1.443 (1.095~1.901)
6.0	4.997	0.025	1.444 (1.046~1.993)
6.5	11.251	0.001	1.773 (1.269~2.477)
7.0	4.830	0.028	1.543 (1.048~2.271)
7.5	7.294	0.007	1.786 (1.173~2.722)
8.0	4.435	0.035	1.725 (1.039~2.866)
8.5	4.214	0.038	1.714 (1.080~2.997)
9.0	4.128	0.040	1.354 (1.026~3.100)
10.0	4.052	0.044	3.236 (1.031~10.154)
11.0	5.137	0.023	5.035 (1.244~20.375)

Abbreviation: HR, hazard ratio; CI, confidence interval.

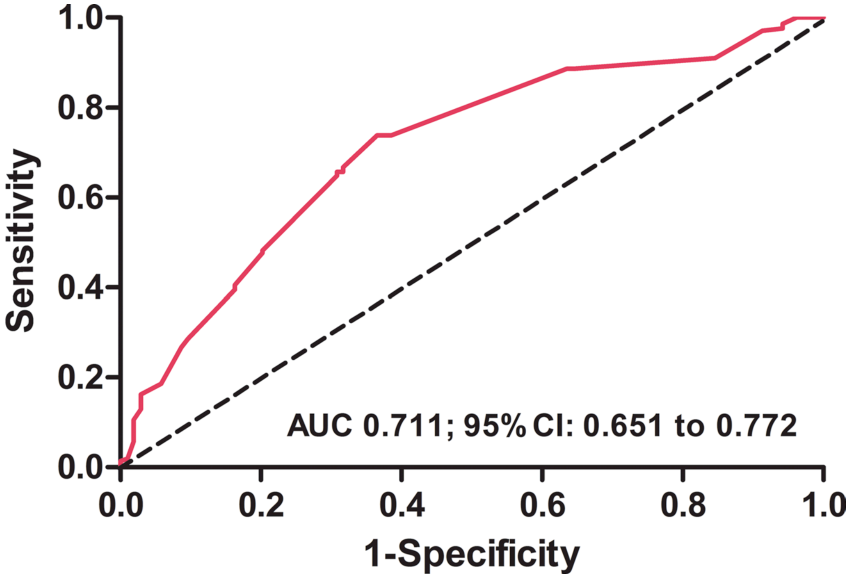

Supplementary Figure S1: ROC curve for the optimal cut-off point of tumor size. The AUC for tumor size was 0.711, with a sensitivity of 73.8% and a specificity of 63.5% by Youden index. The threshold value corresponding to the tumor size was 3.5 cm.
